# Evaluation of recombinant outer membrane protein C based indirect enzyme-linked immunoassay for the detection of *Salmonella* antibodies in poultry

**DOI:** 10.14202/vetworld.2015.1006-1010

**Published:** 2015-08-25

**Authors:** Jinu Manoj, Rajesh K. Agarwal, Blessa Sailo, Mudasir Ahmed Wani, Manoj Kumar Singh

**Affiliations:** 1 Department of Veterinary Public Health and Epidemiology, College of Veterinary & Animal Sciences, Sardar Vallabhbhai Patel University of Agriculture and Technology, Meerut, Uttar Pradesh, India; 2 Division of Bacteriology and Mycology, Indian Veterinary Research Institute, Bareilly, Uttar Pradesh, India; 3 Department of Livestock Production and Management, College of Veterinary & Animal Sciences, Sardar Vallabhbhai Patel University of Agriculture and Technology, Meerut, Uttar Pradesh, India

**Keywords:** antibody, antigen, outer membrane protein, poultry, *Salmonella*

## Abstract

**Aim:**

To evaluate the efficacy of recombinant outer membrane proteinC (rOmpC) based enzyme-linked immunoassay (ELISA) for the diagnosis of salmonellosis in poultry.

**Materials and Methods:**

Three antigens were prepared, and the indirect ELISA was standardized using the antigens and the antiserum raised in chicken against Omp and rOmpC. Sera were collected from a total of 255 apparently healthy field chickens and screened for the presence of *Salmonella* antibodies by this ELISA.

**Results:**

The sodium dodecyl sulfate-polyacrylamide gel electrophoresis analysis of Omp revealed major polypeptides at 36, 42 and 52 kDa, and the rOmpC was evident by a single protein band of 43 kDa. The Omp and rOmpC antigen revealed an optimum concentration of 78 and 156 ng, respectively, in the assay, while the whole cell antigen gave an optimum reaction at a concentration of 10^6^ organisms/ml. The test was found to be specific as it did not react with any of the antisera of seven other organisms. The developed ELISA detected *Salmonella* antibodies from 22 (8.62%) samples with rOmpC antigen, while 24 (9.41%) samples gave a positive reaction with both Omp and whole cell antigens.

**Conclusion:**

We suggest rOmpC based indirect ELISA as a suitable screening tool for serological monitoring of poultry flocks.

## Introduction

Gastrointestinal infections due to *Salmonella enterica* are important from the public health standpoint due to the foodborne nature [1]. Contaminated poultry and poultry products, including eggs are a major source of foodborne *Salmonella* infections [2]. Identification of contaminated poultry hatcheries and infected flocks is important to control and to reduce the transmission of *Salmonella* to susceptible individuals. The routine surveillance of egg layer flocks and broilers is required to detect *Salmonella* infection on a flockwide basis and thus to reduce the potential of contaminated eggs reaching to the consumer [3].

Many diagnostic tests have been developed for the detection of *Salmonella* infections in poultry. Isolation by cultural procedures is the standard and confirmatory method for detection of *Salmonella* in hatcheries and breeding flocks [4]. However, cultural procedures for *Salmonella* detection are laborious, expensive, time consuming and individual birds excrete *Salmonella enterica* intermittently or may eliminate the infection altogether [5]. Thus, the development of reliable screening tests would be useful for the detection of *Salmonella* in hatchery environments and in flocks. Serological methods such as enzyme-linked immunoassay (ELISA) can help in detecting infected and carrier birds as well as silent transmission within the flock, which could be missed by conventional bacteriological isolations due to the intermittent shedding of *Salmonella* [6].

In recent years, outer membrane protein (Omp) has attracted much attention for its potential role as an immunogenic tool in diagnostic assays and vaccination [7-9]. OmpC is one of the major surface antigens with unique exposed epitopes and is expressed in more amounts regardless of the growth condition [10]. OmpC is also reported to be expressed all through the infection period. The hydrophilicity, surface probability and antigenicity calculations of OmpC also confirm the antigenic potential of these regions. The coding gene for OmpC is conserved within different *Salmonella* serotypes [11]. These reasons suggest that OmpC could be a diagnostic antigen because of its specificity, but ELISAs based on Omp for salmonellosis in poultry is not available. The present study was designed to develop an indirect ELISA for the detection of *Salmonella* serovars from field poultry samples.

## Materials and Methods

### Ethical approval

The experiment was conducted after the approval of the Institutional Animal Ethics Committee of Indian Veterinary Research Institute for the raising of antiserum.

### Antigens

Three different antigens *viz*., whole cell antigen, Omp and recombinant Omp C (rOmpC) were prepared and used in this study. *Salmonella* Typhimurium strain E-2375, procured from the repository of the National *Salmonella* Centre (Veterinary), Division of Bacteriology and Mycology, Indian Veterinary Research Institute (IVRI), Izatnagar, India was used for the preparation of antigens. The *Salmonella* strain was tested for their purity, morphological and biochemical characters [12].

The whole cell antigen of *Salmonella* Typhimurium was prepared [13] from brain heart infusion broth (Himedia) culture and adjusted to a density equivalent to McFarland nephlometeric tube No. 4 giving an approximate density of 1200 million bacteria/ml [14] using carbonate-bicarbonate buffer (pH 9.6), followed by heat inactivation at 100°C for 2 h.

The extraction of Omp from *Salmonella* Typhimurium [15] was done. Briefly, *Salmonella* Typhimurium strain (E-2375) was grown in a medium containing 1% tryptone, 1% yeast extract and 0.5% NaCl for 18 h at 37°C with shaking at 200 rpm and pelleted out at 7000 rpm for 30 min using refrigerated centrifuge (Eltek). After washing with normal saline (0.85%), the pelleted culture was resuspended in 30 ml of Tris-HCl buffer (0.1 M; pH 8.0) and sonicated at 10 µ for 10 min and the cell sonicate was centrifuged at 5000 rpm for 10 min at 4°C to remove the cell debris and the supernatant was centrifuged at 30,000 rpm (TH64 rotor Sorval Ultra DuPont) for 90 min at 4°C using sorvall OTD 75B ultracentrifuge (DuPont, USA). The pellet was washed and suspended in phosphate-buffered saline (PBS) (10 mM; pH 7.4).

The rOmpC gene clones expressed in *Escherichia coli* and stabbed in buffered semisolid agar were obtained from the repository of the Veterinary Public Health Division, IVRI, India. The clones were checked by growing on Luria-Bertani agar (Himedia) containing ampicillin and the rOmpC was prepared. Briefly, a loopful of clones was inoculated into 250 ml LB broth containing ampicillin (100 µg/ml) and incubated (Scientronic, New Delhi, India) at 37°C for 24 h. The bacterial cells were harvested after incubation by centrifugation at 6000 rpm for 10 min and the pellet was collected. The polyhistidine tagged rOmpC protein was purified under denaturation condition by Ni-NTA affinity chromatography spin kit (Qiagen, USA). Fifty microlitre of 2× sodium dodecyl ­sulfate-polyacrylamide gel electrophoresis (SDS-PAGE) sample buffer was added to a 50 µl aliquot of each fraction and stored at −20°C for SDS-PAGE analysis for the presence of specific peptide.

### Protein estimation and SDS-PAGE analysis of Omp and rOmpC

The protein content of Omp and rOmpC was analyzed by the NanoDrop^®^ ND-1000 Spectrophotometer (NanoDrop Technologies, Wilmington, DE USA) at 280 nm. SDS (Qualigens, India) polyacrylamide (Ameresco) gel electrophoresis (SDS-PAGE) of all the eluted aliquots was carried out [16] in a vertical SDS-PAGE apparatus (Banglore Genei, India) with 12% acrylamide in separating gel and 5% in stacking gel. The gel was run at 20 mA, stained with 0.25% coomassie brilliant blue R-250 (SRL, India) and destained in 7% glacial acetic acid (SRL, India) solution.

### Hyper immune sera

Hyperimmune serum was raised in chicken against rOmpC and Omp following standard protocol [17]. Briefly, 2 birds of 4 weeks old were used for raising hyper immune sera. Preparations of rOmpC and Omp (200 µg/bird) mixed equally with Freunds incomplete adjuvant (Difco) was injected intramuscularly at multiple sites. Two weeks later, birds were injected with 100 µg/bird of above antigens. Another booster was given at 15 days later. The birds were test bled after 10 days of the booster by cardiac puncture and serum was separated, the antibody titer was determined and stored in cryovials at −20°C for further use. This antiserum was used as positive control in the indirect ELISA.

### Standardization of ELISA

ELISA was standardized [18] by checkerboard analysis using the three antigens and antiserum. Two-fold serial dilutions of rOmpC antigen from 5 µg/0.1ml upto 39.06 ng/0.1ml, were made in carbonate-bicarbonate buffer (pH 9.6). The wells of ELISA plate (Nunc, Denmark) were coated with 100 µl of diluted antigen and incubated at 4°C overnight. After washing 3 times with PBS (10 mM; pH 7.4) containing 0.05% Tween-20 (PBST), wells were blocked with 3% bovine serum albumin (BSA) (Himedia) in PBST for 2 h at 37°C in a moist chamber. With additional three washing with PBST, 100 µl of sera (two-fold serial dilutions of antiserum (from 1:100 to 1:3200) raised in chickens diluted in PBST containing 1% BSA, were added and incubated as above. After washing the plates 3 times, 100 µl of horseradish peroxidase (HRP) labeled rabbit anti-chicken IgY (1:5000, 1:10,000 and 1:15,000 dilutions in PBST with 1% BSA) (Bangalore Genei, India) was added in each well and incubated as above. Finally, after three washes, 100 µl of freshly prepared substrate solution (10 mg O-phenylene diamine (Ameresco) and 1 µl H_2_O_2_ in 10 ml citrate buffer, pH 4.6) was added in each well and incubated in dark for 20 min at room temperature (18-25°C). The color reaction was stopped by adding 100 µl of 1M H_2_SO_4_ and absorbance was measured at 492 nm by ELISA plate reader (Microscan MS5605A, Electronics Corporation of India Ltd.). Antigen control, positive, and negative serum controls along with conjugate control were always included in each plate separately.

The ELISA was similarly standardized for Omp antigen and whole cell antigen also. Omp diluted from 5 µg/0.1ml to 39.06 ng/0.1ml and 10-fold dilutions of the whole cell, ranging from 10^5^ to 10^9^ organisms/ml were used for coating of wells. A positive to negative (P/N) optical density (OD) ratio of >2 was considered as positive in the ELISA test proper.

A comparison of 3% BSA and 5% skim milk powder (SMP) in PBST as a blocking agent was performed with all the three antigens used in this study, after coating the antigens at their corresponding optimum concentrations. Cross-reactivity of the ELISA was also checked by using the three antigens and the antiserum procured from the repository of the National *Salmonella* Centre (Veterinary), Division of Bacteriology and Mycology, IVRI, India, against *Shigella dysenteriae* (E218), *Enterobacter aerogens* (E 220), *Hafnia alvei* (E221), *Proteus morganii* (E225), *Proteus mirabilis* (E226), *Serratia marcescens* (E229), *E. coli* (F2), and *Salmonella* Typhimurium (E2375). The sensitivity of ELISA also checked for 3 months. The plates were coated with rOmpC and Omp antigen at optimum concentration, blocked and stored at 4°C. At weekly intervals, 1 plate was taken out and performed the developed ELISA as per the method described earlier.

### Sample collection and indirect ELISA

A total of 255 poultry blood samples from apparently healthy birds were collected from retail outlets of Bareilly city, Uttar Pradesh, India. Birds were bled through jugular vein and blood (7 ml) was collected in sterile microfuge tubes, allowed to clot and further kept at 4°C overnight for retraction. The serum was separated and collected using Pasteur pipette and stored at −20°C till further use. Indirect ELISA for the detection of *Salmonella* antibodies was performed using the above three antigens and the field poultry serum samples (1:400 dilution) and rabbit anti-chicken IgY-HRP conjugate (1:15,000 dilution).

## Results

### Protein estimation and SDS-PAGE analysis of Omp and rOmpC

**Figure-1:**
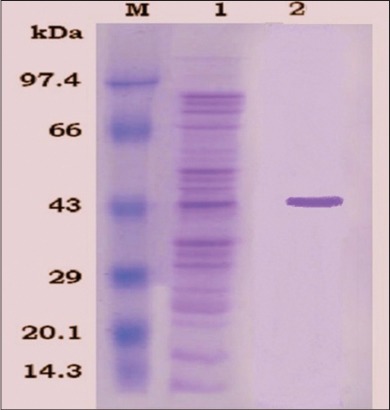
Sodium dodecyl sulfate-polyacrylamide gel electrophoresis analysis, Lane M: Protein molecular weight marker, Lane 1: Outer membrane protein, Lane 2: RecombinantOmp.

The protein content of Omp and rOmpC was found to be 10 and 2.5 mg/ml, respectively. Electrophoretic separation of Omp resolved more than 10 bands after coomassie brilliant blue R-250 staining. The different polypeptides of Omp ranged from 14 kDa to 90 kDa. Out of which 36, 42, and 52 kDa bands were major polypeptides. However, rOmpC revealed a single protein at 43 kDa after purification on SDS-PAGE analysis (Figure-1).

### Optimization of antigen, conjugate and serum

**Figure-2:**
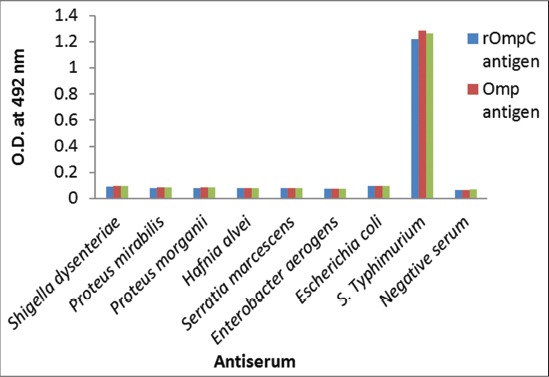
Cross-reactivity studies with various Gram-positive and negative bacteria.

Checker-board titration of Omp and rOmpC antigen revealed an optimum concentration of 78 ng/0.1ml and 156 ng/0.1ml, respectively, while whole cell antigen gave an optimum reaction with a concentration of 10^6^ organisms/ml. These amounts of antigens were used in ELISA test proper. Both the antisera raised in birds gave an optimum reaction at 1:400 dilution. The optimum dilution of rabbit anti-chicken IgY-HRP conjugate was found to be 1:15,000. Comparison between 3% BSA and 5% SMP as blocking agent revealed BSA coated wells with higher P/N ratio than the SMP coated wells with all the three antigens used in the study. Cross-reactivity studies with other bacteria revealed a P/N ratio of ≥2 against *Salmonella* Typhimurium while all other antisera gave a P/N ratio of ≤2 (Figure-2). It was also found that the ELISA was sensitive even upto 3 months of study in the precoated ELISA plates, eventhough the OD value reduced at a very slow rate.

### Indirect ELISA

**Figure-3:**
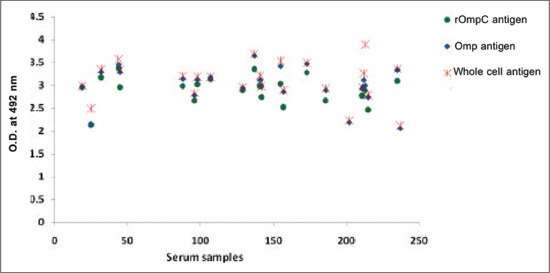
Optical density of positive serum samples.

Out of 255 poultry sera tested, 22 (8.62%) gave a positive reaction with rOmpC antigen and 24 (9.41%) gave a positive reaction with both Omp antigen and whole cell antigen. The OD of positive serum samples ranged from 2.145 to 3.363 with rOmpC antigen, from 2.054 to 3.653 with Omp antigen and from 2.118 to 3.902 with whole cell antigen (Figure-3).

## Discussion

Only a few reports [19] are available regarding Omp based ELISA for the diagnosis of salmonellosis. In this work, Omp extracted from *Salmonella* Typhimurium (E-2375) resulted in an optimum yield of Omp and the SDS-PAGE analysis revealed major polypeptides of 36, 42, and 52 kDa. The rOmpC was also successfully expressed as evident by a single protein band of 43 kDa on SDS-PAGE analysis. The recombinant protein has advantages over whole cell protein as an antigen for the detection of antibodies. The recombinant protein antigen can be prepared without involving the risk of handling the zoonotic microbe; hence it is non-infectious, safe and can be used without containment facilities [20]. Moreover, recombinant protein improves the specificity of the ELISA [21]. This enhanced specificity is especially important for the control and eradication programs in developing countries. The hyper immune sera were raised against Omp and rOmpC in chicken were found to be of high antibody titer, thus avoiding cross reactions by dilution of antiserum [22]. It was evident in the current study, where no cross-reactions were observed at the optimum dilution (1:400) of serum used.

On the basis of checkerboard analysis, the optimum dilution of antigens was found to be 78 ng, 156 ng and 10^6^ organisms/ml for Omp, rOmpC, and whole cell antigens, respectively. In our investigation, it was found that the BSA was a better blocking agent than the SMP. A preliminary assessment of ELISA specificity was also carried out with sera raised against seven other organisms. None of them gave positive results indicating test to be specific. Only homologous antiserum against *Salmonella* Typhimurium gave a P/N ratio of ≥2. Sera from 255 apparently healthy poultry were tested, of which 22 (8.62%) gave positive reaction with rOmpC antigen and 24 (9.41%) gave positive reaction with both Omp antigen and whole cell antigen. Two additional positive results with Omp and whole cell antigens may be due to the presence of additional epitopes, which might have reacted with the *Salmonella* antibodies present in the sera. Researchers have demonstrated that OmpC is the major surface antigen with unique surface exposed epitopes [8]. Even though the three antigens used in the current study gave almost equal results, the recombinant protein would be preferred in practice as it is easier to produce in bulk and as already stated it might also improve the specificity of the test. Since OmpC is a genus conserved gene, the rOmpC based ELISA will possibly be able to detect all *Salmonella* serotypes. Indirect ELISA developed in this study holds promise as screening of poultry flocks for *Salmonella* infection.

## Conclusion

Omps are considered effective antigens to stimulate immune responses because they are exposed on the bacterial surface and easily recognized by the host immune system Therefore, OmpC seems to hold promise as a diagnostic antigen against avian salmonellosis caused by different serovars. The present study evaluated the efficacy of OmpC as a diagnostic tool against *Salmonella* in poultry.

## Authors’ Contributions

JM and RKA designed the study and planned the research experiment. JM performed the research experiments. BS, MAW and MKS helped in collection of samples and processing them. All authors read and approved the final manuscript.
